# Congenital malaria with atypical presentation: A case report from low transmission area in India

**DOI:** 10.1186/1475-2875-6-43

**Published:** 2007-04-13

**Authors:** Neena Valecha, Sunita Bhatia, Sadhna Mehta, Sukla Biswas, Aditya P Dash

**Affiliations:** 1 National Institute of Malaria Research, 22-Sham Nath Marg, Delhi – 110 054, India; 2 Department of Paediatrics, Kasturba Hospital, Darya Ganj, Delhi – 110 002, India

## Abstract

**Background:**

Malaria during first few months of life may be due to transplacental transfer of parasitized maternal erythrocytes. Although IgG and IgM antimalarial antibodies can be detected in maternal blood, only IgG antibodies are present in the infant's blood. These antibodies can delay and modify the onset of clinical manifestations.

**Case Presentation:**

An infant is described who presented with irritability and feeding problems. Clinical examination and investigations revealed that the infant was afebrile, had jaundice, hepatosplenomegaly and haemolytic anaemia. Peripheral smear demonstrated *Plasmodium vivax*. While the mother had significant levels of immunoglobulin G (IgG), the infant was found negative for IgG and had low immunoglobulin M (IgM) levels. The mother had a history of febrile illness during pregnancy and her peripheral smear was also positive for *P. vivax*. Both were successfully treated with chloroquine in the dose of 25 mg/kg/day over three days.

**Conclusion:**

The case emphasizes the importance of considering the diagnosis of malaria even in infants in low transmission area, who may not present with typical symptoms of malaria, such as fever, but have other clinical manifestations like jaundice and haemolytic anaemia.

## Background

Congenital malaria can be acquired by transmission of parasites from mother to child during pregnancy or perinataly during labour [1]. Congenital malaria has been documented for many years but it was previously thought to be uncommon specially in indigenous populations [2]. More recent studies, however, suggest that incidence has increased and values between 0.3 to 33% have been observed from both endemic and non-endemic areas [2]. Mukthar et al. [3] attribute the higher incidence to an increased resistance and virulence of parasite resulting from altered antigenic determinants in addition to increased reporting. Although placental infections with *Plasmodium falciparum *have been described, the role of other species especially *Plasmodium vivax*, which exists in good proportion in some countries, including India, is not known. In a study conducted in central India although 2.2% (4/182) women were placenta positive for *P. vivax *or mixed infection, but none of the infants developed parasitaemia up to 6 months of age [4].

Children with congenital malaria can present with fever, irritability, feeding problems, hepatosplenomegaly, anaemia and jaundice [5]. A case of congenital malaria is reported in a six weeks old male infant who presented with irritability, decreased feeding and jaundice for four days.

The case demonstrates the need of considering congenital malaria as differential diagnosis even in low endemic areas, especially in countries where there is social practice of moving the pregnant woman to her native place, which may be endemic for malaria, for child birth.

## Case presentation

The infant weighing 2.4 kg was a full-term, normal hospital delivery born to non-consanguineous parents. There was no history of physiological jaundice or any other parental problem. The child was on exclusive breast feeding since birth. On physical examination, the infant was afebrile and showed marked pallor and icterus. There was no dehydration. Hepatosplenomegaly was present (liver 4 cm, spleen 4 cm below costal margin). There was no ascites and other systems were normal. The infant was, therefore, investigated for severe anaemia with jaundice and hepatosplenomegaly. Laboratory tests revealed anaemia (Hb 6 gm%) with normal WBC and platelet counts.

The reticulocycte count was 8%. Peripheral smear demonstrated moderate anisocytosis, dimorphic red blood cells with predominate microcytosis and hypochromia. Serum bilirubin was 10 mg/dL (conjugated 4.6 and unconjugated 5.4), SGOT 92 IU units/l and SGPT 100 IU units/l and alkaline phosphatase was 23.6 KA. X-ray chest and ultrasound were normal except hepatosplenomegaly. Blood studies for Toxoplasma, Rubella, Cytomegalovirus and Herpes (TORCH) were negative. Haemoglobin chromatography was normal. Twenty-four hours after admission Hb decreased to 4.5 gm% and repeat peripheral smears were examined more intensively for haemolysis. Smears were found to be positive for *P. vivax *and were referred to the National Institute of Malaria Research, where the presence *P. vivax *trophozoites with a parasitaemia of 0.1% (5000/μl) was confirmed.

Packed cells transfusion was given and the infant was treated successfully with chloroquine at a dose of 25 mg/kg over three days. He was discharged after four days and at time of discharge his Hb was 9 gm%. Follow up of patient was again done after 10 days, when he had no clinical jaundice, his spleen was 2 cm below costal margin and he was accepting feeds. Four weeks later liver functions, haemoglobin (10.5 gm%) and other investigations were normal.

Since patient reported from an area of very low malaria transmission (with an average annual parasite incidence of last five years of 0.08), the possibility of a new infection is remote. A directed history revealed that the mother had, during 7^th ^month of pregnancy, suffered febrile illness in her native place, which has a low to moderate endemicity for malaria. Although exact history of intake of antimalarials is not clear, she improved gradually. During the present episode she had no fever, but her peripheral smear showed presence of scanty *P. vivax *trophozoites suggesting the possibility of congenital malaria in the infant.

Other causes of haemolytic anaemia in infants were excluded and there was no ABO/RH incompatibility in parents and G6PD was normal. Antibody profile of both mother and child sera were also tested for IgG and IgM isotypes against PvMSP1 peptide and *P. vivax*-infected erythrocyte lysate (PvL). The mother's serum showed substantial amount of IgG antibody to both the antigens. While significant level of IgM to these antigens was found in her serum, the serum of the infant found negative for IgG with both the antigens, whereas a very low level of IgM antibody with PvL was detected.

## Conclusion

Although malaria continues to be major public health problem in India and pregnant women have been described as vulnerable group, there have been very few epidemiological studies to determine disease burden in pregnancy and infants [6]. As such, congenital malaria which is acquired by transmission of parasitized maternal erythrocytes across the placenta due to microdamage occurs infrequently in endemic areas. The majority of studies from Africa have focused on falciparum malaria. Congenital malaria in *P. vivax *has been described from Sri Lanka [7], Italy [8], Singapore [9] and Thailand [10].

In the present case, which had atypical presentation with no fever, diagnostic work up was done to rule out the other causes for haemolytic anaemia, jaundice and hepatosplenomegaly. The presence of parasitaemia in the low transmission period in a non-endemic area led us to label the case as congenital malaria which was further substantiated by history of travel in mother. Thus the epidemiological and circumstantial evidence strongly supports transplacental route of infection.

It has been reported that clinical symptoms are rarer in younger infants and absence of febrile episodes has been described [11]. This has been attributed to transplacentally acquired antibodies (IgG), which confer transient protection to infant and thus manifestations are mild. Although IgG and IgM antimalarial antibodies can be detected in maternal blood, only IgG is normally found in the infant's blood. Asymptomatic presentation has also been described by Hindi et al [12]. In the present case, although fever was not present, other severe manifestations were seen, perhaps due to failure of maternal IgG transfer to the infant. In addition, a delay in diagnosis due to non-specific presentation may have contributed to severity.

The treatment with chloroquine was adequate since there is no exoerythrocytic life cycle in congenitally-acquired vivax infection. The report suggests differential diagnosis of congenital malaria should be considered in areas where it is least suspected due to low transmission of disease and also emphasizes importance of history of travel of mother. Emphasis should also be given to awareness regarding use of chemoprophylaxis and bed nets in community and adequate treatment for suspect malaria during antenatal period.

## Authors' contributions

SB and SM were responsible for clinical management of the patient admitted in their hospital. NV was involved in investigation for congenital malaria and writing the manuscript. SB carried out the immunoassays. AP was involved in revising the manuscript and giving final approval of the version.
